# Randomised, double-blind, clinical investigation to compare orlistat 60 milligram and a customized polyglucosamine, two treatment methods for the management of overweight and obesity

**DOI:** 10.1186/s40608-016-0130-4

**Published:** 2017-01-11

**Authors:** Manfred Stoll, Norman Bitterlich, Umberto Cornelli

**Affiliations:** 1 Diabetological Center, Frankfurter Str. 50, D-63303 Dreieich, Germany; 2 Medizin and Service GmbH, Abt. Biostatistik, Boettcherstr. 10, D-09117 Chemnitz, Germany; 3 Loyola University School of Medicine, 2160 South First Avenue, Maywood, Illinois 60153 USA

**Keywords:** Polyglucosamine, L 112, Overweight, Obesity, Orlistat, Weight reduction, Weight loss

## Abstract

**Background:**

The efficacy of a non-prescription drug to support weight loss programs has yet to be compared. This clinical trial investigates the comparability of orlistat 60 milligram (mg) and polyglucosamine.

**Methods:**

Sixty-four overweight or obese subjects were included in a two-center double-blind study. One center was in Germany [center 1] and the other was in Italy [center 2].

The subjects (26 in center 1 and 38 in center 2) were recommended to follow a calorie deficit of about 2000 kilojoules/day and to increase their physical activity to 3 metabolic equivalent hours (MET h)/day. In both centers, subjects were randomized to receive polyglucosamine (2 tablets x 2) or orlistat (1 capsule x 3) for a period of 12 weeks. Weight loss was considered as a main variable together with the reduction of 5 per cent (%) of body weight (5R). Body Mass Index (BMI) and waist circumference (WC) were taken as secondary variables.

**Results:**

A significant difference in weight loss between the two groups was shown, 6.7 ± 3.14 kilogram (kg) in group polyglucosamine versus 4.8 ± 2.24 kg in group orlistat (t test *p* < 0.05) respectively; BMI and WC reduction were also more consistent with polyglucosamine treatment than with orlistat treatment (t test *p* < 0.05). No significant difference was found in the number of subjects who achieved 5R (70% for polyglucosamine and 55% for orlistat group; chi square *p* > 0.05).

The administration of polyglucosamine following energy restriction and increase in physical activity reduces body weight, BMI and WC more efficiently than orlistat

**Conclusions:**

Even though both groups were instructed to adopt a calorie restricted diet together with increased physical activity an additional weight loss in the polyglucosamine group of 1.6 kilogram (kg) compared to the orlistat group (6.2 ± 3.46 versus 4.6 ± 2.36 kg) in both centers was seen despite the higher consumption of carbohydrates in Italy (center 2). A typical Italian diet is usually high in carbohydrate content whereas Germans tend to consume meals with higher fat content. This leads to the assumption that polyglucosamine limits both fat and carbohydrate absorption which would explain the comparable effective weight reduction in the Italian participants.

**Trial registration:**

Trial registration at ClinicalTrials.gov NCT02529631, registered on Aug 19, 2015 retrospectively registered.

## Background

Overweight and obesity are major public health challenges of the 21st century in the European region [1] and guidelines to assist practitioners and patients for an appropriate treatment have been compiled by many professional societies for nutrition [2]. Therapists often recommend the use of weight loss aids such as orlistat to obtain a more rapid weight loss due to the ability of this product to inhibit the pancreatic lipase and the dietary triglycerides bioavailability [3].

The withdrawal of registered weight loss products from the market has led therapists to look for currently available treatment options. One product that is also used to help support body weight management is polyglucosamine, a low molecular weight chitosan (LMWC) that binds fats, creating an emulsion that [4] makes them non-bioavailable. The emulsion can be partially eliminated or used by colonic bacteria as a fuel due to their ability to hydrolize LMWC with the bacterial enzyme chitosanase [5, 6].

For both products to obtain a reduction in body weight of about 5% in a relatively short period of time (2 to 4 months), a daily caloric restriction combined with increased physical activity is recommended.

There are currently no studies comparing the two products and there exist no published data in the literature. The aim of this study was to compare their effectiveness in a double blind clinical trial in two different centers.

## Methods

### Trial design

The trial was a randomized, double-blind study in two centers comparing the treatment effects of orlistat and polyglucosamine and conducted in accordance with the European Medical Device Directive 92/43/EEC, European harmonized Standard (EN) International Standardization Organisation (ISO) 14155-1, the Declaration of Helsinki and the National Data Protection Act. The centers involved in the study were: center 1, the Diabetological center in Dreieich-Sprendlingen, Germany, center 2, the Monitoring Food and Diseases (MAP) in Rende (Cosenza, Italy).

### Participants

**Fig. 1:**
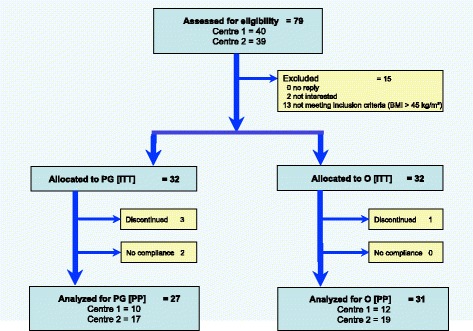
CONSORT Statement Flow Chart

Sixty four subject were admitted (26 in center 1 and 38 in center 2) as shown in Fig. 1.

Patient recruitment and development during the randomized double-blind clinical investigation comparing polyglucosamine and orlistat.

The admission criteria were overweight subjects with a BMI ≥ 28 kilogram/square-meter (kg/m^2^) and < 45 kg/m^2^; waist circumference of more than 80 centimeter (cm) for women and greater than 94 cm for men; age 21 to 70 years.

The energy intake was also an important admission criterion. The kilojoule (kJ) intake was measured using a questionnaire based on weekly servings [7] and those subjects reporting an energy intake lower than the standard value calculated according to Miffin St-Jeor Equation (based upon weight, height, age) [8] were excluded from the clinical trial.

Other exclusion criteria were as follows*:* pregnancy or breast-feeding, addiction to alcohol, inability to fulfill the requirements of the trial protocol, cancer, malignant tumor, hypersensitivity reactions to crustaceans or any of the ingredients of the two products. Patients with chronic disease not brought under control with an appropriate therapy or with diabetes were excluded.

All patients were informed in detail about the purpose of the clinical trial both orally and in writing and their written consent obtained. Insurance to cover the participants, at a level appropriate to the risks posed by the clinical trial was provided and complied with the principles of the latest version of the Declaration of Helsinki (October 2008).

All patients were given the same instructions regarding dietary changes based on the requirements outlined in a nutrition course manual, which includes a list of foods to be avoided (or reduced) in order to achieve 2000 kJ/day deficit (about 500 kilocalories (kcal)). Those foods high in energy density such as processed meat (sausages, salami etc.), meat, cheese, butter, oil, pasta, beer, wine / alcohol, sweet beverages were particularly cautioned against overconsumption.

All patients were taught how to increase physical activity level at intensity equivalent to 3 METs/day and given a fitness digital versatile disk (DVD) featuring an exercise program to help motivate them to continue doing exercises on their own. The recommendation of expending 3 METs/day corresponds to 21 METs/week (about 1 hour/day of moderate intensity exercise) and was based upon the cut-off to prevent weight gain while consuming a usual diet [9].

The energy expenditure of 3 METs corresponds approximately to 45 minutes (min) of walking or 15 min of biking at 15 kilometer per hour (km/h), or 15 min of swimming [10].

### Variables

The primary target variable was the body weight, whereas the other anthropometric measures (BMI and waist circumference) were considered secondary variables only.

The cutoff reduction of 5% of body weight (5R) was also taken as a primary goal.

The plasma lipids and blood pressure were also measured but they were not considered as variables because patients under therapy with antihypertensive drugs and/or statins were also admitted to the trial.

All the measurements were taken at the moment of the enrolment (Visit 1/T1) and at least four times during the therapy: at baseline, after 4, 8 and 12 weeks of treatment.

### Investigational medical device and comparator

Orlistat 60 mg (1 capsule x 3) was filled in blue capsules and polyglucosamine (2 tablets x 2) was available as compressed pale colored tablets. However, there was a difference in the dosing regimens: 3 x 1 capsule (a capsule three times daily with each meal) and 2 x 2 tablets (two tablets twice a day with a meal).

Push-through blisters, each containing 3 x 1 blue capsules and 3 x 2 ivory colored tablets were given to both treatment groups. Therefore, these patients were each given 2 tablets and a capsule three times a day.

All participants received the same number of tablets and capsules (see Table 1).

**Table 1:** Treatment scheme; double blind placebo/polyglucosamine/orlistat

Either		
Breakfast	2 placebo tablets	1 placebo capsule
Lunch	2 polyglucosamine tablets	1 placebo capsule
Dinner	2 polyglucosamine tablets	1 placebo capsule
Or		
Breakfast	2 placebo tablets	1 orlistat 60 mg capsule
Lunch	2 placebo tablets	1 orlistat 60 mg capsule
Dinner	2 placebo tablets	1 orlistat 60 mg capsule

In the group treated with polyglucosamine, two tablets, also called placebo tablets (provided for breakfast) contained no active substance

Double Dummy Design blister pack

**Fig. 2:**
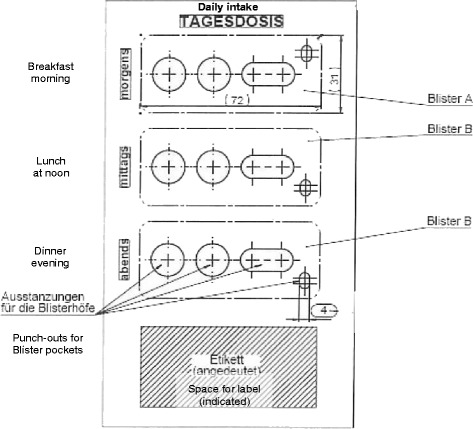
Double Dummy Design blister pack

Thirty-two blister packs each providing one-day supply (6 tablets + 3 capsules) were given to study subjects so that every four weeks they had to return to the center for a new supply. The subjects were requested to attend the follow-up visits by phone calls (see Fig. 2).

### Sample size

A sample size of 40 patients in each group had a sufficiently high probability (Cohen's effect size 0.5, 5% significance level, 80% power and 20% drop out) of detecting a statistically significant difference by means of the t-test. The sample was not stratified by gender. For the random process, block randomization was used with a block of size four.

### Compliance

Measurement of medication adherence was obtained by counting the number of residual tablets. The compliance was fixed to a consumption of at least 44 blisters during the study period (48 blisters were given and 46 should have been consumed). The physical activity and the caloric restriction were not taken as a compliance measure.

### Statistical methods

The metric data were characterized according to their statistical parameters, mean value, standard deviation and extrema. The differences between the groups were calculated by means of the t-test (probability (p)-value p_t_) under the assumption that the variances were the same. The correlation coefficient (r) was calculated between the initial body weight and the body weight reduction.

For the evaluation of the primary endpoints, the results of the intention to treat analysis (ITT) were compared to those of per-protocol population (PP). For the analysis of subjects reaching 5R the Chi^2^-test was used. The biometric analysis was performed using the statistical software package SPSS®, Version 19.0 and Microsoft Excel® was used to add new data records to a list and create a graphic illustration of the results.

## Results

According to the randomisation list 32 subjects (50%) were assigned to the polyglucosamine treatment and another 32 subjects (50%) were allocated to the orlistat treatment.

Fifty-eight subjects concluded the trial, 27 in the polyglucosamine group and 31 in the orlistat group, respectively. In the ITT population, 6 patients were excluded from the analysis of the PP population.

-Four subjects reported side effects: 3 in the polyglucosamine group (meteorism, constipation and vomiting) and one in the orlistat group (diarrhea):

Group polyglucosamine:

- Patient No. 7 (discontinued after visit 8) because of meteorism
- Patient No. 12 (discontinued after visit 4) because of constipation
- Patient No. 14 (discontinued after visit 4) because of nausea and vomiting

Group orlistat:

- Patient No. 34 (discontinued after visit 2) because of diarrhoea.

Two subjects of the polyglucosamine group were excluded because the compliance was lower than 95% (about 75% and 80%, respectively), whereas all the subjects in the orlistat group were compliant.

The complaints given as the reason for the termination in group polyglucosamine were symptoms such as stomach ache and bloating, nausea and vomiting as well as constipation, palpitations and mood swings. Medical treatment was not sought for these complaints as they were only temporary and without any further consequences. As a result of stress and an irregular lifestyle including occasional diarrhoea, discontinuation of the treatment in the orlistat group took place after the second visit, as requested by the patients. All the other recorded adverse events/reactions were mild and transient and medical attention was not required. The adverse events /reactions occurred with a similar frequency in both treatment groups. The symptoms were consistent with those specified in the respective patient information leaflet. The occurrence of serious adverse events (SAE) was not observed in both regimens.

The anthropometric measurements recorded at baseline were similar in both groups (see Table 2).

**Table 2:** Anthropometric measures at baseline (ITT: number (*N*) = 64) in groups to be treated with polyglucosamine and orlistat

Variable	Total	Group polyglucosamine	Group orlistat	p^a^/^b^
N	64	32	32	
Gender (male/female)	28/36	16/16	12/20	P = 0.313^a^
Age (years)	50.0 ± 9.17	50.0 ± 9.10	50.1 ± 9.38	P = 0.989^b^
Weight (kg)	99.4 ± 12.33	100.6 ± 13.22	98.2 ± 11.47	P = 0.446^b^
Height (m)	169.3 ± 8.09	170.3 ± 7.60	168.4 ± 8.58	P = 0.358^b^
BMI (kg/m^2^)	34.7 ± 4.21	34.6 ± 3.70	34.8 ± 4.73	P = 0.896^b^
WC (cm)	111.2 ± 10.66	112.4 ± 10.95	110.0 ± 10.38	P = 0.358^b^

^a^Chi square test; ^b^t test

There were no significant changes in blood pressure, pulse rate and laboratory findings between the two treatment groups (data not reported). Hence, both treatment methods can be considered to be comparable in efficacy for these last variables.

The average modifications of the anthropometric variables are reported in Tables 3, 4, 5 and 6.

**Table 3:** Anthropometric measurements (PP) at different control times (T1 baseline and, T5, T9, T13) in groups treated with polyglucosamine and orlistat

Variable	Group	T1^a^	T4^a^	T9^a^	T13^a^
Weight (kg)	polyglucosamine	100.9 ± 13.44	97.2 ± 12.61	95.4 ± 12.79	94.1 ± 13.41
	orlistat	97.9 ± 11.55	95.1 ± 11.24	94.5 ± 11.98	93.1 ± 11.82
BMI (kg/m^2^)	polyglucosamine	34.6 ± 3.69	33.4 ± 3.58	32.8 ± 3.50	32.3 ± 3.59
	orlistat	34.7 ± 4.76	33.7 ± 4.60	33.4 ± 4.68	33.0 ± 4.63
WC (cm)	polyglucosamine	113.4 ± 11.13	109.6 ± 12.10	107.4 ± 11.85	105.1 ± 11.98
	orlistat	109.5 ± 10.13	106.8 ± 9.55	104.2 ± 9.30	103.4 ± 9.14

^a^The differences between groups are not statistically significant (t test)

**Table 4:** Anthropometric measurements (PP) at different control times (T1 baseline and, T5, T9, T13) in groups treated with PG and O

Variable	Group	T1	T1 - T5	T1 - T9	T1 - T13
Weight (kg)	polyglucosamine	100.9 ± 13.44	3.71 ± 2.67	5.49 ± 2.63#	6.74 ± 3.14#
	orlistat	97.9 ± 11.55	2.82 ± 1.42	3.43 ± 1.69	4.78 ± 2.24
BMI (kg/m^2^)	polyglucosamine	34.6 ± 3.69	1.26 ± 0.88	1.89 ± 0.90#	2.33 ± 1.09*
	orlistat	34.7 ± 4.76	1.00 ± 0.54	1.23 ± 0.64	1.71 ± 0.86
WC (cm)	polyglucosamine	113.4 ± 11.13	3.81 ± 3.11	5.96 ± 4.13	8.33 ± 4.42*
	orlistat	109.5 ± 10.13	2.61 ± 2.65	5.29 ± 2.47	6.10 ± 3.43

#*p*<0.01, * *p* < 0.05

**Table 5:** Anthropometric measurements (ITT) at different control times (T1 baseline and, T5, T9, T13) in groups treated with PG and O

Variable	Group	T1^a^	T4^a^	T9^a^	T13^a^
Weight (kg)	polyglucosamine	100.6 ± 13.22	97.2 ± 12.37	95.6 ± 12.54	94.3 ± 13.06
	orlisat	98.2 ± 11.47	95.5 ± 11.25	94.9 ± 11.99	93.6 ± 11.88
BMI (kg/m^2^)	polyglucosamine	34.6 ± 3.70	33.5 ± 3.62	32.9 ± 3.61	32.5 ± 3.69
	orlistat	34.8 ± 4.73	33.8 ± 4.60	33.6 ± 4.69	33.1 ± 4.66
WC (cm)	polyglucosamine	112.4 ± 10.95	109.2 ± 11.46	107.4 ± 11.25	105.1 ± 11.21
	orlistat	110.0 ± 10.38	107.4 ± 9.98	104.8 ± 9.93	104.1 ± 9.84

^a^The differences between groups are not statistically significant (t test)

**Table 6:** Anthropometric measurements (ITT) at different control times (T1 baseline and, T5, T9, T13) in groups treated with PG and O

Variable	Group	T1	T1 - T5	T1 - T9	T1 - T13
Weight (kg)	polyglucosamine	100.6 ± 13.22	3.36 ± 2.66	4.98 ± 2.90#	6.24 ± 3.46*
	orlistat	98.2 ± 11.47	2.73 ± 1.48	3.32 ± 1.77	4.63 ± 2.36
BMI (kg/m^2^)	polyglucosamine	34.6 ± 3.70	1.13 ± 0.88	1.71 ± 0.99*	2.15 ± 1.21
	orlistat	34.8 ± 4.73	0.97 ± 0.54	1.20 ± 0.67	1.66 ± 0.90
WC (cm)	polyglucosamine	112.4 ± 10.95	3.22 ± 3.29	5.06 ± 4.56	7.34 ± 4.83
	orlistat	110.0 ± 10.38	2.53 ± 2.65	5.12 ± 2.60	5.91 ± 3.54

#*p* <0.01, *p < 0.05

At visit (T)1, the average value of the body weight in the polyglucosamine group was higher than in the orlistat group (3.0 kg), but the difference was not statistically significant (t test; *p* > 0.05).

During the 12-week period, there was a reduction in all the anthropometric variables for both treatment groups (Table 3). However, the reduction of all the variables (Table 7) was significantly more consistent in the group treated with polyglucosamine.

**Table 7:** Body weight decrease following the treatment with polyglucosamine and orlistat

Decrease in body weight [kg]	N	Average ± standard-deviation(SD)	Mini- mum	Maxi- mum	Cut off 5% decrease [N]	Cut off 5% decrease [%]
Total (PP)	58	5.7 ± 2.85	0.3	13.0	36	62.1
Group polyglucosamine	27	6.7 ± 3.14	0.3	13.0	19	70.4
Group orlistat	31	4.8 ± 2.24	1.5	11.9	17	54.8
P t value	0.008				Chi square	p > 0.05
Total (ITT)	64	5.4 ± 3.05	-0.9	13.0	37	57.8
Group polyglucosamine	32	6.2 ± 3.46	-0.9	13.0	21	65.6
Group orlistat	32	4.6 ± 2.36	0.0	11.9	16	50.0
Pt value	0.033				Chi square	p > 0.05

**Fig. 3:**
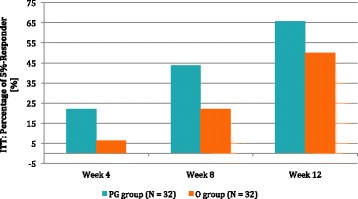
ITT percentage of 5-%- Responder, is the percentage of subjects with a body weight reduction of at least 5% compared to baseline

**Fig. 4:**
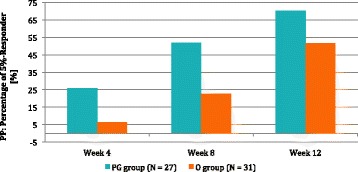
PP percentage of 5-%-Responder, is the percentage of subjects with a body weight reduction of at least 5% compared to baseline

**Fig. 5:**
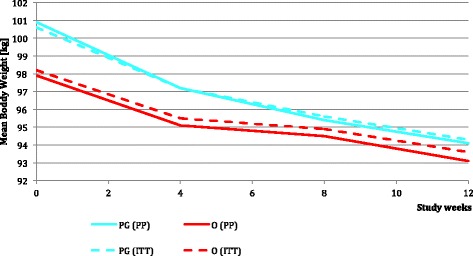
Comparison of the mean body weight in kg

The average body weight reduction within the 12-week period (T13-T1) for PG was significantly higher for both the ITT and PP analyses (see Figs. 3, 4 and 5).

The reduction of BMI was -2.3 ± 1.09 kg/m^2^ in the group polyglucosamine and -1.7 ± 0.86 kg/m^2^ in the group orlistat; the WC modification was also more pronounced following the polyglucosamine treatment than with orlistat, -8.3 ± 4.42 cm and -6.1 ± 3.43 cm, respectively.

The differences were statistically significant (*t* test *p* < 0.05) both for ITT and PP analyses, with the only exception for WC in the ITT analysis where the difference between the two groups turned out not to be statistically significant (*t* test *p* = 0.179).

The number of subjects that reached 5R was not different in the two groups (Table 7) even though after treatment, there was an increase in percentage for both the ITT and PP analyses (see Table 7).

There was no significant correlation between weight reduction and weight at baseline (*r* = 0.101 in the ITT and *r* = 0.104 in the PP). However, BMI measurements obtained in center 1 were more favorable (1.46 versus 1.40 in center 1, 2.14 versus 1.13 in center 2). Gender did not affect the results.

The data mentioned below are the general outcomes using repeated measures ANOVA. In fact, if the curves of the changes do not intersect with each other, a significant outcome during the course can be expected when there are significant differences across time points. Therefore, we can conclude that the results are valid.

PP: Taking into account weight loss over time during the four visits V1, V5, V9 and V13, the factor time (F-test: 157.3; p_time_ < 0.001) as well as the group differences over time (F-test: 6.2; p_time x group_ = 0.002) show a statistically significance (see Table 8).

**Table 8:** Weight loss over time in the PP group

Body weight PP	Sum of squares (Type III)	Degree of freedom df	F-test	p-value
Factor Time	1054.662	3	157.285	<0.001
Time x Group	41.360	3	6.167	0.002
Error	375.551	168	-	-

ITT: Taking into account weight loss over time during the four visits V1, V5, V9 and V13, the factor time (F-test: 139.5; ptime <0.001) as well as the group differences over time (F-test: 4.2; ptime x group= 0.017) show a statistical difference (see Tables 9 and 10).

**Table 9:** Weight loss over time in the ITT group

Body weight ITT	Sum of squares (Type III)	df	F-test	p-value
Factor Time	1033.924	3	139.545	<0.001
Time x Group	31.234	3	4.216	0.017
Error	459.374	186	-	-

**Table 10:** Results of the separate analysis of the data reported

Parameter	Factor Time		Time x Group	
	F-test	*p*-value	F-test	*p*-value
Body weight (PP)	157.285	<0.001	6.167	0.002
Body weight (ITT)	139.545	<0.001	4.216	0.017
BMI (PP)	153.830	<0.001	5.027	0.007
BMI (ITT)	136.927	<0.001	3.306	0.041
Waist circumference (PP)	125.351	<0.001	2.831	0.059
Waist circumference (ITT)	105.392	<0.001	1.568	0.213

The p-values were determined using the Greenhouse-Geisser correction

The results obtained from the separate analysis of data reported in center 1 (Germany) and center 2 (Italy) were slightly different.

In center 1, the two products ended up with similar body weight reduction in the PP analysis (-4.9 ± 4.18 kg for polyglucosamine and -5.3 ± 3.03 kg for orlistat, respectively); in center 2, the body weight reduction was more consistent for polyglucosamine than for orlistat (-7.8 ± 1.73 kg and -4.5 ± 1.58 kg, respectively).

The development of weight loss in the two groups is shown in Fig. 5.

The red solid line shows the reduction in body weight (kg) of the orlistat 60 mg group (PP). The red dashed line shows the reduction in body (kg) weight of the orlistat 60mg group (ITT). The turquoise solid line shows the reduction in body weight (kg) of the polyglucosamine group (PP). The turquoise dashed line shows the reduction in body weight (kg) of the polyglucosamine group (ITT).

## Discussion

The purpose of this clinical trial was to conduct a direct comparison between two treatments, orlistat and polyglucosamine as a treatment option for body weight management.

Orlistat is used worldwide in obese and overweight subjects and is one of the most commonly used weight loss medications in Europe for weight management according to the labelling text approved by the European Medicines Agency [11].

There are some clinical studies with orlistat at 60 mg and 120 mg against placebo [3, 12–14] in subjects undergoing caloric restriction for a period of treatment ranging between 14 and 104 weeks. These trials show that in general, an approximate weight reduction of about 2 kg can be added to weight loss induced by caloric restriction alone.

There are also studies on polyglucosamine reporting a similar or an even higher weight reduction [4, 15] in subjects following caloric restriction and treated for a period of time ranking between 12 and 24 weeks.

A recent study found that in a large number of cases (115 subjects comparing polyglucosamine versus Placebo) the consumption of polyglucosamine plus energy restriction of about 2000 kJ combined with an increase in physical activity level to 7 METs/week for 24 weeks induced a reduction of 4.5 kg [16].

In the present study, the intensity of physical activity was increased to 21 METs/week and the body weight reduction was more evident despite a shorter period of treatment. Following this schedule, the mean weight loss in both regimens, regardless of gender and the initial body weight, was a reduction of more than 4 kg body weight in 12 weeks. These results confirm the importance of adding more physical activity to any type of pharmacological treatments.

Similar recommendations for diet and physical exercise are part of the current guidelines of international societies for nutrition, obesity, and diabetes.

However, a particular aspect has to be considered in relation to the more consistent effect shown in center 2.

This center is located in South of Italy where the carbohydrate consumption, in terms of bread and pasta, is more common than in Germany.

Pasta in particular has to be addressed, because in Italy its consumption is about 80 gram/day/person. An “average” dish of pasta consists of at least 1500 kJ and the intake of most of the overweight pasta consumers frequently exceeds 2500 kJ / portion [17]. Despite the different ingredients used to prepare a dish of pasta (oil, cheese, meat etc.), the energy content is mainly due to carbohydrates (75-80%) than to fats and proteins. This implies that a limitation of the energy intake of 2000 kJ/day in the subjects enrolled in center 2 was derived mainly from carbohydrates [18–20], whereas in center 1 (Germany) the caloric restriction was mainly derived from a reduction in dietary fat (sausages, meat, butter).

In other terms, the energy intake restriction was identical in the two centers but the type of food to be avoided was not identical.

The bioavailability of fats is reduced by both polyglucosamine (fat emulsion effect) and by orlistat (lipase inhibition). However, from experimental data on polyglucosamine an increase of glucose in faeces was found [6] also indicating a reduction of carbohydrate availability. This last aspect has been shown indirectly during the therapy of metabolic syndrome, where the polyglucosamine treatment was found to reduce blood glucose levels as well [4].

In theory, whereas orlistat limits the fat bioavailability, polyglucosamine seems to limit both fat and carbohydrate absorption and this difference gives a reasonable explanation for the similar effective weight reduction in a diet with carbohydrates as the main energy source.

## Conclusion

In conclusion, there are indications that the more evident effect of polyglucosamine compared to orlistat on the anthropometric variables could be determined by the quality of energy limitation (carbohydrates/fats).

Although more data should be provided in this area to confirm our observations, the results of the current trial give an insight to the different outcomes that can be obtained with the same product in different countries characterized by different food cultures.

## Abbreviations

%: Per cent
5R: Reduction of 5% of body weight compared to the initial body weight
BfArM: Federal Institute for Drugs and Medical Devices
BMI: Body Mass Index kg/m^2^
cm: Centimeter
df: Degree of freedom
DVD: Digital versatile disk
EN: European standard prepared in the technical committees of the European Committee for Standardization CEN
h: Hour
ISO: International Standardization Organisation
ITT: Intention to treat
Kcal: Kilocalories
kg: Kilogram
kJ: kilojoule
km: Kilometre
km/h: Kilometer per hour
LMWC: Low molecular weight chitosan
MET h: Metabolic equivalent hours
mg: milligram
min: Minute
n / N: Number
No.: Number
Orlistat: Tetrahydrolipstatin
Polyglucosamine: ß-1,4-polymer of D-glucosamin and N-acetyl-D-glucosamine
PP: Per protocol
SAE: Serious adverse events
SD: Standard deviation
T1: 1st visit
T13: 13th visit
WC: Waist circumference
